# Adipose transplantation improves olfactory function and neurogenesis via PKCα-involved lipid metabolism in Seipin Knockout mice

**DOI:** 10.1186/s13287-023-03463-9

**Published:** 2023-09-07

**Authors:** Jing Yang, Na Yang, Huifang Zhao, Yan Qiao, Yanqiu Li, Chunfang Wang, Kah-Leong Lim, Chengwu Zhang, Wulin Yang, Li Lu

**Affiliations:** 1 Department of Anatomy, Shanxi Medical University, Taiyuan, 030001 People’s Republic of China; 2 School of Basic Medical Sciences, Shanxi Medical University, Taiyuan, 030001 People’s Republic of China; 3 Analytical Instrumentation Center and State Key Laboratory of Coal Conversion, Institute of Coal Chemistry, Chinese Academy of Sciences, Taiyuan, People’s Republic of China; 4 Laboratory Animal Research Center of Shanxi Medical University, Shanxi Key Laboratory of Animal and Animal Model of Human Diseases, Shanxi Medical University, Taiyuan, People’s Republic of China; 5 Lee Kong Chian School of Medicine, Nanyang Technological University, Singapore, Singapore; 6 Anhui Province Key Laboratory of Medical Physics and Technology, Center of Medical Physics and Technology, Hefei Institutes of Physical Science, Chinese Academy of Sciences, Hefei, 230031 People’s Republic of China; 7 Cancer Hospital, Hefei Institutes of Physical Science, Chinese Academy of Sciences, Hefei, People’s Republic of China; 8 Key Laboratory of Cellular Physiology, Ministry of Education, Shanxi Medical University, Taiyuan, People’s Republic of China

**Keywords:** Lipid metabolism, Subventricular zone, Neurogenesis, Adipose tissue transplantation, Protein kinase C α

## Abstract

**Background:**

Lipodystrophy-associated metabolic disorders caused by Seipin deficiency lead to not only severe lipodystrophy but also neurological disorders. However, the underlying mechanism of Seipin deficiency-induced neuropathy is not well elucidated, and the possible restorative strategy needs to be explored.

**Methods:**

In the present study, we used Seipin knockout (KO) mice, combined with transcriptome analysis, mass spectrometry imaging, neurobehavior test, and cellular and molecular assay to investigate the systemic lipid metabolic abnormalities in lipodystrophic mice model and their effects on adult neurogenesis in the subventricular zone (SVZ) and olfactory function. After subcutaneous adipose tissue (AT) transplantation, metabolic and neurological function was measured in Seipin KO mice to clarify whether restoring lipid metabolic homeostasis would improve neurobehavior.

**Results:**

It was found that Seipin KO mice presented the ectopic accumulation of lipids in the lateral ventricle, accompanied by decreased neurogenesis in adult SVZ, diminished new neuron formation in the olfactory bulb, and impaired olfactory-related memory. Transcriptome analysis showed that the differentially expressed genes (DEGs) in SVZ of adult Seipin KO mice were significantly enriched in lipid metabolism. Mass spectrometry imaging showed that the levels of glycerophospholipid and diglyceride (DG) were significantly increased. Furthermore, we found that AT transplantation rescued the abnormality of peripheral metabolism in Seipin KO mice and ameliorated the ectopic lipid accumulation, concomitant with restoration of the SVZ neurogenesis and olfactory function. Mechanistically, PKCα expression was up-regulated in SVZ tissues of Seipin KO mice, which may be a potential mediator between lipid dysregulation and neurological disorder. DG analogue (Dic8) can up-regulate PKCα and inhibit the proliferation and differentiation of neural stem cells (NSCs) in vitro, while PKCα inhibitor can block this effect.

**Conclusion:**

This study demonstrates that Seipin deficiency can lead to systemic lipid disorder with concomitant SVZ neurogenesis and impaired olfactory memory. However, AT restores lipid homeostasis and neurogenesis. PKCα is a key mediator mediating Seipin KO-induced abnormal lipid metabolism and impaired neurogenesis in the SVZ, and inhibition of PKCα can restore the impaired neurogenesis. This work reveals the underlying mechanism of Seipin deficiency-induced neurological dysfunction and provides new ideas for the treatment of neurological dysfunction caused by metabolic disorders.

**Supplementary Information:**

The online version contains supplementary material available at 10.1186/s13287-023-03463-9.

## Introduction

Lipid metabolism is the process involving the synthesis and degradation of lipids such as phospholipids, glycolipids, sphingolipids, cholesterol, prostaglandins, and diglyceride [1]. According to Yang et al. (2022), aberrant lipid metabolism is implicated in pathogenesis of various disorders [1, 2]. Seipin is the critical mediator of lipid biogenesis and metabolism, which is highly expressed in the adipose tissue (AT) and the brain [3]. Seipin deficiency leads to onset of type 2 congenital generalized lipodystrophy (CGL2), which is characterized by the absence of whole-body AT and metabolic disorders such as hepatic steatosis, insulin resistance (IR), dyslipidemia as well as mental retardation [3, 4]. Systemic Seipin knockout (KO) mice displayed abnormal brain development, impaired spatial memory, and depression, which could be mimicked by neuronal specific Seipin KO [5–7]. Those results indicate that Seipin might play a critical role in the central nervous system, while the mechanism underlying memory failure in Seipin deficiency remains unclear.

It has been proved that the intelligence and mental development is closely related to neurogenesis [8]. Neurogenesis occurs mainly in two brain regions, the subgranular layer of the dentate gyrus in the hippocampus and the subventricular zone (SVZ) of the lateral ventricle [8]. Unlike the dentate gyrus, NSCs in the SVZ extend a minute apical ending to contact the ventricle and a long basal process ending on blood vessels, enabling them to exchange materials directly with the cerebrospinal fluid and bloodstream [9]. In addition, NSCs residing in SVZ continually generate new neurons that integrate into preexisting circuits and participate in cognitive functions such as perceptual learning and olfactory memory [10]. Lipid biosynthesis and fatty acid metabolism genes are highly expressed in NSCs of SVZ, which suggests that lipid metabolism might affect the neurogenesis of NSCs and the neuronal function [11]. Aberrant lipid metabolism is apt to render neuropathy. Indeed, obese mice showed the accumulation of lipid droplets (LD) in SVZ, which impairs NSCs differentiation [12]. Accumulation of oleic acid-rich triglycerides was found in the SVZ of 3xTg-AD mice as well as AD patients, directly affecting NSCs activity [13]. These results suggest that lipid metabolism disorders may lead to neurogenesis impairment in SVZ, and there may be a common underlying mechanism among them [14]. Whether and how Seipin deficiency-induced lipid metabolism abnormality affects the neurogenesis of NSCs and neurobehavior needs to be verified, and a possible strategy to rescue the phenotypes is to be explored.

In this study, we used Seipin KO mice with systemic lipid dysregulation as a model to investigate the association between lipid metabolism disorders and SVZ neurogenesis and olfactory dysfunction. Next, we revealed the potential mechanisms and intervention strategies. It was found that olfactory-related memory significantly declined in adult Seipin KO mice. Consistently, the proliferation and differentiation of NSCs were reduced considerably in adult Seipin KO mice. In addition, lipid ectopic accumulation in the SVZ region was observed in adult Seipin KO mice, including diacylglycerol (DG), phosphatidic acid (PA), phosphatidylcholine (PC), phosphatidylethanolamine (PE), and triglyceride (TG). Noteworthy, subcutaneous AT transplantation restored lipid metabolic homeostasis and neurobehavior dysregulation of Seipin KO mice. Mechanistically, protein kinase C (PKC) α was found to be a key mediator of Seipin deficiency-induced lipid metabolism and neurological dysregulation. Expression of PKCα was up-regulated in SVZ of Seipin KO mice. Treatment of cultured NSCs with DG analogue Dic8 could also lead to up-regulation of PKCα and compromise the proliferation and differentiation of NSCs, while Go6983, an inhibitor of PKCα, could reverse those phenotypes. The present study elucidated the underlying mechanism with which lipid metabolism disorders led to neurobehavioral abnormalities and revealed that AT transplantation could be one feasible way for the intervention of lipid metabolism disorders. It provides clues for therapeutic of the neurological diseases caused by dysregulation of lipid metabolism.

## Materials and methods

### Mice

C57/BL6 mice were obtained from the Animal Center of Shanxi Medical University and Seipin heterozygous mice (Seipin^±^) were generously donated by Prof. Han Weiping (Agency for Science, Technology and Research, Singapore). All animal studies were performed in accordance with the Institutional guidelines approved by the Animal Research Ethics Committee of Shanxi Medical University. Seipin^±^ mice were bred and inter-crossed to obtain homozygous knockout mice along with C57/BL6 wild type (WT) mice. All mice were kept in an animal facility maintained at 22℃ ± 2℃ under a 12 h light/dark cycle. Food and water were available ad libitum.

### NSCs culture and DG treatment

Primary NSCs cultures were prepared from SVZ of newborn Seipin KO and WT mice euthanized by cervical cord dislocation following the protocol established previously [15]. Briefly, cells were plated at 1 × 10^5^ cells/mL and cultured in DMEM-F12 proliferation medium supplemented with 2% B27 (Invitrogen, Stockholm, Sweden), 20 ng/mL epidermal growth factor (EGF; PeroTech, Rocky Hill, NJ, USA), and 20 ng/mL basic fibroblast growth factor (bFGF; PeroTech) in a humidified incubator at 37 °C with 5% CO_2_. After a week of culture, neurospheres were gained and digested with Accutase Cell Detachment Solution (Gibco) to obtain single cell suspension. Then NSCs were plated onto the coverslips pre-coated with 50 μg/ mL poly-L-ornithine and 20 μg/mL laminin (Sigma-Aldrich, Saint Louis, MO, USA) overnight. To measure the effect of DG on NSCs proliferation, cells were treated with 50 μM and 100 µM DiC8 (Aladdin Chemistry Co, Shanghai, China) or PKC inhibitor (Go6983; 140 nM; MedChemExpress, Monmouth Junction, NJ, USA) and cultured in DMEM supplemented with BrdU (10 μM) for 12 h for immunofluorescence analysis. For NSCs differentiation, the culture medium was replaced with differentiation medium containing 1% fetal bovine serum, 1% B27 supplement, DiC8, and/or Go6983. After three days, cells were harvested for immunostaining.

### Brain dissection and tissue processing

Mice were anesthetized by inhalation of isoflurane (Abbott, Baar, Switzerland) and then fixed by transcardiac perfusion with 4% paraformaldehyde (PFA). Brains were carefully dissected and post-fixed overnight at 4 °C. After dehydration in 30% sucrose, OCT (Sakura, Tokyo, Japan) embedded brains were serially sliced into 16-μm-thick sections using a cryostat (Leica Microsystems, Wetzlar, Germany). One of every six consecutive sections encompassing the SVZ (bregma 1.09 mm to 0.13 mm) or the olfactory bulb (bregma 4.57 mm to 4.07 mm) was selected for immunostaining.

### Immunofluorescence staining

For immunofluorescent staining, 4% PFA-fixed sections or cells were permeabilized with 0.3% Triton X-100 (Sigma-Aldrich) in PBS for 15–20 min and blocked with 10% goat serum for 1 h at room temperature (RT). The specimens were incubated with primary antibodies overnight at 4℃. The primary antibodies used in the present study are listed in Table 1. Goat anti-rabbit IgG H & L (Alexa Fluor 555), Goat anti-mouse IgG H & L (Alexa Fluor 555), and Goat anti-mouse IgG H & L (Alexa Fluor 488) were used as appropriate secondary antibodies and incubated for 1 h at RT. Nuclei were counterstained with 4,6-diamidino-2-phenylinedole, dihydrochloride (DAPI; Sigma-Aldrich) at 1 μg/mL for 5 min.

**Table 1:** Antibodies used in this study

Antibodies	Species	Application	Dilution	Company and Catalog number
Rabbit anti-DCX	Rabbit	IF	1:200	Cell Signaling technology, Cat# 4604
Rabbit anti-Ki67	Rabbit	IF	1:400	Abcam, Cat# ab15580
Rabbit anti-NeuN	Rabbit	IF	1:400	Proteintech Group, Cat# 26975-1-AP
Mouse anti-BrdU	Mouse	IF	1:200	Abclonal, Cat# A1482
Mouse anti-Tuj1	Mouse	IF	1:300	Sigma-Aldrich, Cat# MAB1637
Goat anti-rabbit IgG H & L (Alexa Fluor 555)	Rabbit	IF	1:200	Invitrogen, Cat# A-21429
Goat anti-mouse IgG H & L (Alexa Fluor 488)	Mouse	IF	1:200	Invitrogen, Cat# A-28175
Goat anti-mouse IgG H & L (Alexa Fluor 555)	Mouse	IF	1:200	Invitrogen, Cat# A-21424
Mouse anti-PKCα	Mouse	WB	1:1000	Santa Cruz, Cat# sc-8393
Mouse anti-β-actin	Mouse	WB	1:4000	Sigma-Aldrich, Cat# A2228
Rabbit IgG, HRP	Rabbit	WB	1:5000	ZSGB-BIO, Cat# ZB-2306
Mouse IgG, HRP	Mouse	WB	1:5000	ZSGB-BIO, Cat# ZB-2305

Sections for BrdU staining were denatured to expose antigen with 2 N HCl at 37 °C for 10–15 min in tissues before the Triton X-100 treatment. The sections are then incubated successively with anti-BrdU antibody overnight at 4 °C. Then, Alexa Fluor 488 goat anti-mouse IgG for 1 h at RT. The fluorescence images were captured by Olympus BX51 microscope (Olympus, Tokyo, Japan) or Leica SP8 confocal microscope (Leica Microsystems).

### Quantitation of fluorescent images

The fluorescent images were analyzed in a double-blinded manner using MetaMorph software (Molecular Devices, Sunnyvale, CA, USA) from at least three independent experiments. Serial sections from comparable positions were used to count positive cells in vivo, as reported previously [16]. For each mouse, 6 sections were counted and the average number of Ki67^+^ and DCX^+^ cells in each area was calculated, then multiplied by the number of sections per SVZ to obtain the total number per mouse. For the olfactory bulb, 6 sections were taken and the average number of BrdU^+^/NeuN^+^ cells was calculated. In vitro, for each coverslip, six different non-overlapping regions were randomly selected at 20-fold magnification and the percentage of BrdU^+^ and Tuj1^+^ cells were calculated.

### Olfactory habituation/dishabituation test

To assess the olfactory bulb function, olfactory habituation/dishabituation tests were performed. WT mice were age matched from the same litters as the Seipin KO mice. Before the test, each mouse was placed in the chamber (31 × 25 × 12.5 cm) for 30 min to acclimate new environment. For each test, the odors were detected in the following order: deionized water, isoamyl acetate (IAA), vanilla (1 mg/mL), female mice urine and male mice urine, and the testes were repeated three times, at 1 min intervals. Successive trials with different odors were separated by 5 min. The duration of sniffing behavior toward each odor stimulus was recorded.

### Olfactory short-term memory text

The olfactory short-term memory test was conducted to assess the ability of mice to recognize familiar odors. Mice habituated to experimental conditions during the adaptation phase were placed in a 31 × 25 × 12.5 cm chamber. Mice were subjected to odorants dropped on filter paper (2 × 2 cm) for 5 min at 30, 60, 90, or 120 min intervals. A different odor was used at each interval point, but each mouse was tested at only one interval per day to avoid cross interference of olfactory detection and memory. If mice remembered the odor from the 1st trial, they were expected to spend less time sniffing when subjected to odorant for the 2nd time.

### Olfactory avoidance test

Olfactory avoidance behavior to non-dehydrogenated 2, 4, 5-trimethylthiazole (nTMT), a synthetic fox feces odor, was recorded to assess mice olfactory sensitivity. During the adaptation phase, each mouse was acclimated in a cage of the same size as the test cage (31 × 25 × 12.5 cm) for 30 min, then transferred to a new test cage for 30 min, and repeated four times. During the test phase, the test cage was divided into two equal areas and then nTMT was added to the filter paper (2 × 2 cm) on the one side of the test cage at the concentration of 0%, 6%, and 12%, respectively. The mice were placed in the middle of the test cage, and the time they stayed on the other side of the cage was observed and recorded for 10 min.

### Oil Red O staining

The frozen SVZ sections mounted on superfrost glass slides were allowed to air dry for 12 h at RT. After air drying, the slides were placed in 60% isopropanol for 1–3 s and stained with Oil Red O solution (Sigma-Aldrich) for 10 min. Next, the sections were washed with distilled water, rinsed with 60% isopropanol for 1 min, and washed for another 5 min. The staining of slides was imaged using a microscope (Olympus). The area and intensity of LD in the SVZ ventricle wall were quantified by Adobe Photoshop software, and normalized by the lateral ventricle perimeter in each image. The intensity was quantified in three different subregions of SVZ, a depth of 20 μm from the ventricular wall.

### AT transplantation

AT transplantation was performed on mice at one-month-old, and littermates were used to avoid rejection. Subcutaneous AT of euthanized donor mice was taken and cut into 1 cubic centimeter pieces. WT or Seipin KO mice were randomly divided into transplantation group and sham group. Then, the mice were anesthetized with 2.5% isoflurane (Abbott) and a small incision (1 cm^3^) was created in the back. The graft (500–900 mg) was inserted through a syringe into the subcutaneous space through the incision. Sham-operated control mice received the same operation but without adipose injection. After surgery, incisions were closed using 4–0 silk sutures. When the mice were fully awake, they were sent back to the animal room for feeding and observation. AT transplantation was performed once a month and continued for 4 months. During this period, the liver/body weight was recorded. Before killing, the AT was evaluated visually by IRIS positron emission tomography/computed tomography (IRIS PET/CT; Inviscan SAS, Strasbourg, France). The HU value of the adipose tissue was between -300 and -100. After the identification of AT, the region-growing thresholding algorithm was used to estimate AT volume with a very bright adipose signal by OsiriX imaging software (OsiriX Foundation, Geneva, Switzerland). Adipose weight was calculated by multiplying the adipose volume and the adipose density. For histological evaluation of transplanted tissue, AT was removed 4 months after transplantation. Following formalin fixation, AT was paraffin-embedded, and 4 μm paraffin sections were stained with hematoxylin and eosin (HE).

### HE staining

The fresh dissected AT was fixed with a fixator for more than 24 h and subjected to alcohol gradient dehydration. Then, the wax-soaked AT was embedded and placed in the paraffin slicer for sectioning to a 4 μm slice. The paraffin sections were dewaxed and dyed with hematoxylin solution (Servicebio, Wuhan, China) for 3–5 min. Hematoxylin differentiation solution (Servicebio) was differentiated for 2–5 s, and the Hematoxylin Scott Tap Bluing (Servicebio) was treated for 2–5 s. The slices were dyed in eosin solution (Servicebio) for 5 min and then placed in gradient alcohol and xylene for 5 min, respectively, and sealed with neutral gum. Lastly, the tissue sections were observed under the Olympus BX51 microscope (Olympus) and the images were collected for analysis.

### Calculation of liver-to-Body weight ratio

Body weight and wet liver weight were recorded immediately after the experimental animals were killed. The liver-to-body weight ratio was calculated using the formula: liver weight (g) × 100% / body weight (g).

### Determination of plasma adiponectin and leptin levels

Serum was collected after 1000 rpm centrifugation. Adiponectin and leptin levels were measured using a specific ELISA kit (Cloud-Clone Corp, Wuhan, China). Sample dilution and standard calibrators were prepared following kit instructions. Briefly, ELISA plate coated with capture antibody was incubated with 100 μL test samples or standard at 37℃ for 2 h, and then, biotinylated antibody was added and incubated at 37℃ for 1 h. After washing away the unbound biotinylated antibody, the HRP labeled avidin was added. Tetramethylbenzidine substrate was added following an additional washing step. The absorbance (OD value) was measured at 450 nm wavelength excitation using a microplate reader (BioTek Instruments, Winooski, VT, USA) to calculate the sample concentration.

### Glucose and insulin tolerance tests

For glucose tolerance assay, mice were intraperitoneally injected with 2 mg/kg of glucose after 6 h fasting. Blood samples were collected from the tail vein. Glucose values were measured at 0, 15, 30, 60, 90, and 120 min post-injection with a glucometer (Johnson & Johnson, New Brunswick, NJ, USA). For insulin tolerance assay, mice were injected intraperitoneally with 0.75 mIU/g insulin after 4 h fasting, and glucose levels were measured at 0, 15, 30, 60, and 90 min post-injection.

### Matrix-assisted laser desorption ionization time-of-flight mass spectrometry (MALDI-TOF MS) analysis

The SVZs of mice were dissected on ice and placed in the tissue-homogenizing tube, which was frozen in liquid nitrogen. The frozen tissue block was sliced into 12-μm-thick sections by cryostat microtome (Leica Microsystems) and thaw-mounted onto indium tin oxide (ITO)-coated glass slides. After the above slices into ITO were dried, they were sprayed with O-P, N–C/G solution as the matrix (1 mg/mL) by using an automated spraying device (HTX Technologies LLC, Carrboro, NC, USA) and dried thoroughly in a vacuum desiccator [17]. Then, the SVZs tissues were identified by MALDI-TOF MS and all analyses were performed with an UltrafleXtreme mass spectrometer (Bruker Daltonics, Bremen, Germany). The imaging software conducted the MALDI-TOF MS data from three different subregions of the lateral ventricles at a depth of 20 μm surrounding the ventricular wall. Two-dimensional ion density maps for different compound mass-to-charge ratios (m/z) were obtained after smoothing and baseline calibration with SCiLS Lab 2020a software. Significantly different m/z values (*p* < 0.05) between the two groups were obtained based on ROC analysis and t tests in SCiLS Lab 2020a software.

### RNA isolation and transcriptome analysis

The total RNA of SVZ was isolated by TRIzol reagent (Molecular Research Center, Cincinnati, Ohio, USA) according to the manufacturer’s instructions. Transcriptome sequencing and data analysis were completed by Biomarker Technologies Company (Beijing, China). Hierarchical cluster analysis based on the differentially expressed genes (DEGs) was filtered with *p* value < 0.05, and log2 fold change (Log2 FC) > 0.5 or < –0.5 in each pairwise comparison. The Gene Ontology (GO) enrichment and the Kyoto Encyclopedia of Genes and Genomes (KEGG) were analyzed with KOBAS software.

### Western blotting

Cells or brain tissues were washed with ice-cold PBS and lysed in RIPA lysis buffer supplemented with the protease inhibitor cocktail (Thermo Fisher Scientific, Pittsburgh, PA, USA). Total protein concentration was determined using the BCA Protein Assay Kit (Beyotime Biotechnology). An equal amount of proteins were separated by 10% SDS–polyacrylamide gels and transferred to polyvinylidene difluoride membranes (Millipore, Billerica, MA, USA). The membrane was blocked in 5% milk in TBST (TBS containing 0.1% Tween-20) for 1 h at RT and incubated with primary antibodies overnight at 4℃. After washing with TBST, the membrane was incubated with horseradish-coupled secondary antibody for 2 h at RT. Immunoreactive protein bands were visualized using an ECL detection kit (Thermal Biotech, Rockford, IL, USA). The band intensities were quantified using Image-J software, and densitometry values were normalized to the corresponding β-actin density. Antibodies utilized here are listed in Table 1.

### Statistical analysis

No statistical tests were used to predetermine sample sizes; however, sample sizes are consistent with similar published literature [15, 16]. Mice were randomly assigned to experimental time points or groups, and no animals or data points were excluded from analyses. Investigators were blinded to group allocation during data collection and analysis. All experiments were repeated at least three times. All statistical analyses were performed using Prism software (GraphPad Software, San Diego, CA) and presented as mean ± standard deviations (SD). The unpaired Student’s t test was used for comparisons between two groups, and two-way ANOVA with Tukey’s post hoc test was used for multi-group comparisons. The* p* value < 0.05 was selected to determine statistical significance.

## Results

### Seipin KO impaired olfactory-related memory and SVZ neurogenesis of adult mice

**Fig. 1:**
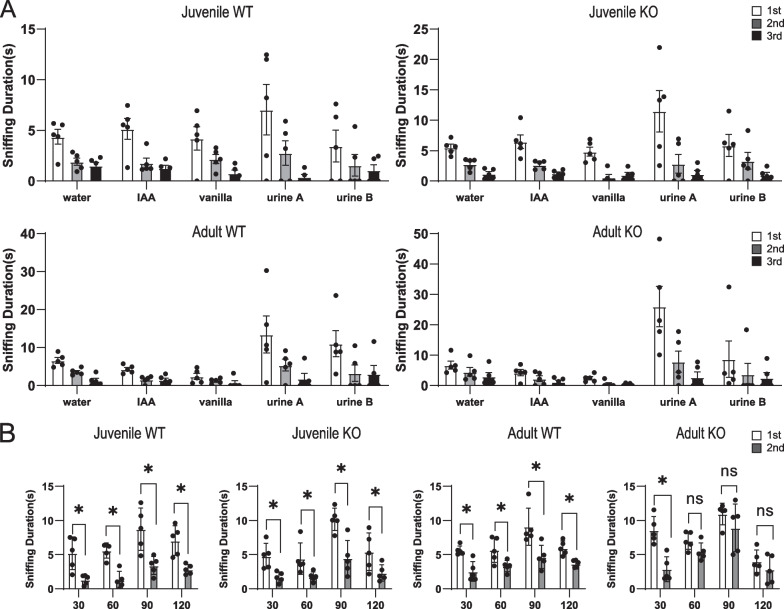
Olfactory function assay of Seipin KO and WT mice. **A** Time spent investigating water, IAA, vanilla, female mice urine, and male mice urine (n = 5). **B** The sniffing duration for mice presented with the same odorants twice at the indicated time intervals (n = 5). Data were shown as means ± SD. *p < 0.05

**Fig. 2:**
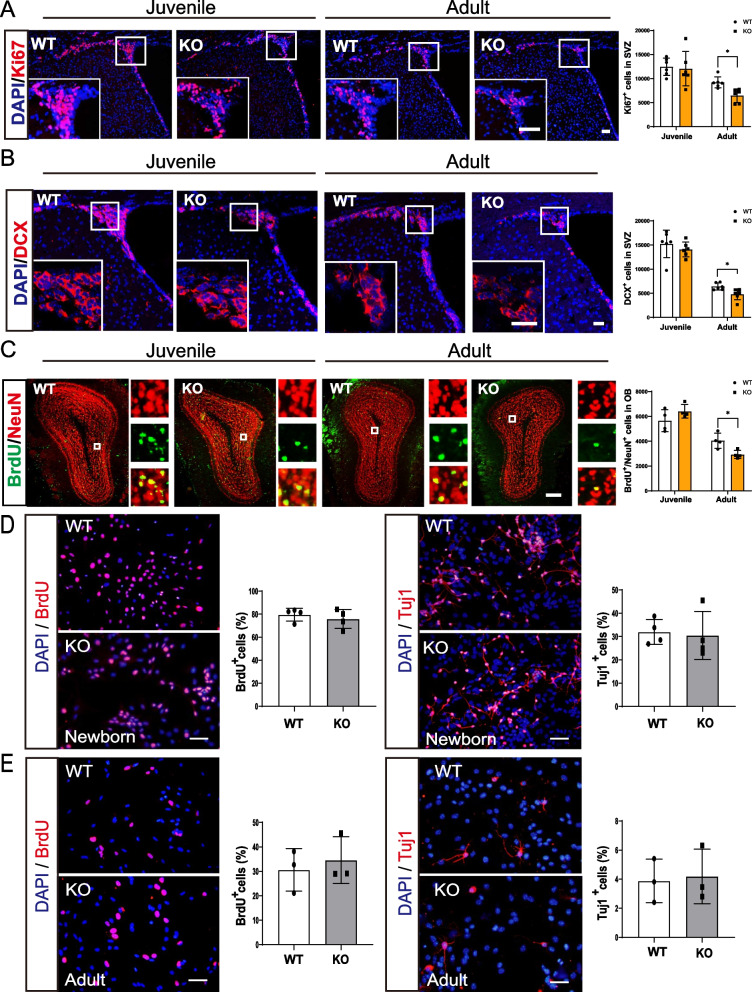
SVZ neurogenesis in adult Seipin KO mice. **A**–**C** Immunofluorescence analysis for Ki67, DCX, and BrdU/NeuN in SVZ (**A** and **B**) and olfactory bulb (**C**) of juvenile and adult WT and Seipin KO mice, respectively. The right panel corresponded to a higher magnification view of the boxed region shown in the merged image. n = 6 (**A** and **B**), Scale bar = 50 μm in (**A**), Scale bar = 25 μm in (**B**); n = 4, Scale bar = 200 μm in (**C**). **D** and **E** The proliferation and differentiation capacity of cells as determined by BrdU staining and Tuj1 staining in NSCs from neonatal (**D**) and adult mice (**E**), respectively. DAPI was used as a nuclear counterstain. Scale bar = 50 μm in (D and E). Data were shown as means ± SD. *p < 0.05 vs. adult WT

It has been established that aberrant lipid metabolism impairs neurogenesis and neurobehavior [13]. We firstly examined the olfactory function in Seipin KO mice. The odor habituation/dishabituation test showed that either juvenile (1-month-old) or adult (5-month-old) Seipin KO mice could sense odorants (Fig. 1A). For the short-term olfactory memory measurement, mice were presented with the same odorant twice with an interval between the two presentations. Generally, mice showed less interest in odorants that they had encountered previously. Compared with the first presentation, the sniffing duration of the second exposure was significantly decreased at four intervals (30, 60, 90, and 120 min) in juvenile Seipin KO mice. However, adult Seipin KO mice exhibited a similar interest at the 60 min, 90 min, and 120 min intervals, indicating that olfactory-related memory capacity was impaired in the adult stage (Fig. 1B). Considering that adult SVZ neurogenesis is necessary for olfactory memory, NSCs proliferation and differentiation were measured by Ki67 and DCX staining. As displayed in Fig. 2A and B, the number of Ki67^+^ cells and DCX^+^ neurons in SVZ of adult Seipin KO mice was significantly decreased, but not that of juvenile one. Likely, the number of BrdU^+^/NeuN^+^ cells in the olfactory bulb of adult Seipin KO mice was considerably lower than that of control, further confirming the impairment of SVZ neurogenesis in adult Seipin KO mice (Fig. 2C). To clarify that the observed deficit in SVZ neurogenesis of Seipin KO mice is systemic effect or NSCs intrinsic only, we carried out in vitro NSCs culture. It showed that the number of BrdU^+^ proliferating cells and Tuj1^+^ neurons in cultured NSCs from neonatal and adult Seipin KO mice did not show significant difference compared with that from WT control (Fig. 2D and E). It indicated that compromised neurogenesis and olfactory-related memory in adult Seipin KO mice might be rendered by systemic or microenvironment rather than the intrinsic loss of Seipin in NSCs.

### Dysregulated lipid metabolism in SVZ region of adult Seipin KO mice

**Fig. 3:**
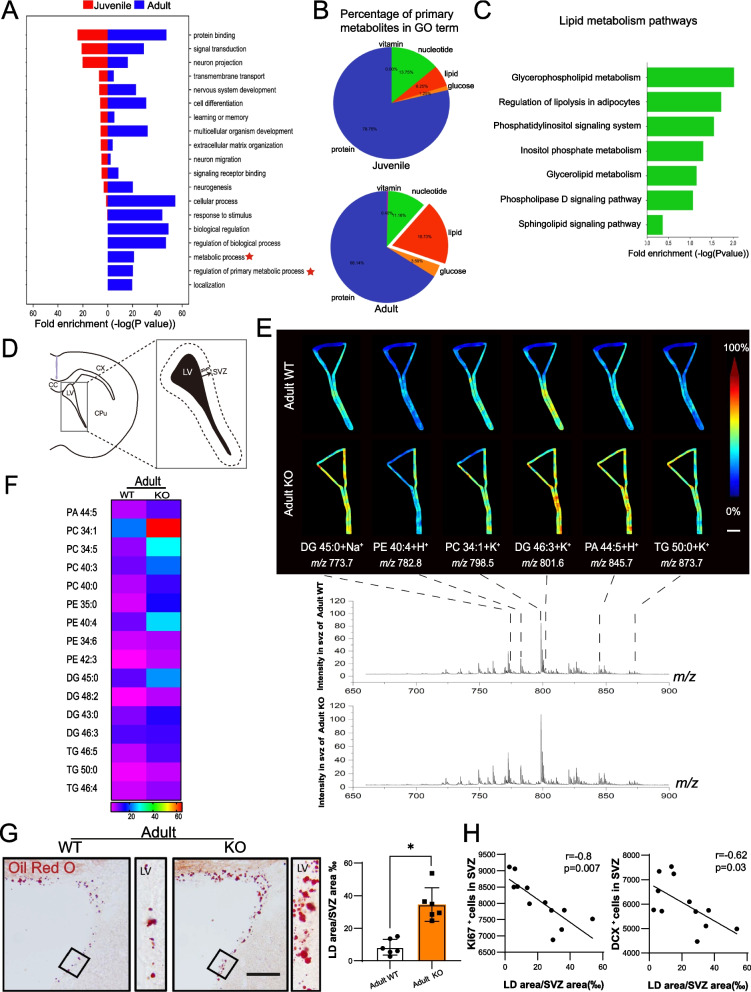
Altered lipid metabolism in the SVZ of adult mice. **A** GO analysis of genes in SVZ of Seipin KO and WT mice. **B** Pie charts showing the percentage of lipid metabolism in SVZ primary metabolism of adult and juvenile Seipin KO mice. **C** KEGG pathway analysis showing the lipid metabolism-related DEGs, including glycerophospholipid, phospholipase D, and sphingolipid signaling pathways between adult Seipin KO and WT mice. **D** Pattern diagram of SVZ region by MALDI-TOF MS analysis. CX: cortex, CPu: caudate putamen, CC: corpus callosum, LV: lateral ventricle. **E**: MALDI-TOF MS showing significantly accumulated lipids in the SVZ of adult Seipin KO mice and their representative m/z ratio peaks. Scale bar = 100 μm. **F** Heatmap analysis showing the most significantly accumulated lipid components in the SVZ of adult Seipin KO mice (n = 3). **G**: Oil Red O detection of LD in the SVZ of the adult brain of Seipin KO mice (n = 6), Scale bar = 50 μm. **H** Pearson correlations analyses between neurogenesis impairment and the area of LD accumulation (n = 6). Data were shown as means ± SD. *p < 0.05 vs. adult WT

After observing the impact of Seipin deficiency on the NSCs of SVZ, we wish to know the mechanism underlying neurogenic disorders in adult Seipin KO mice. The high-throughput transcriptome analysis on SVZ tissues of Seipin KO mice was performed. As shown in Fig. 3A and B, DEGs were significantly enriched in metabolism-related biological processes in adult Seipin KO mice compared with juvenile ones. Further analysis revealed that significant alterations concentrated in glycerophospholipid metabolism, phosphatidylinositol, and sphingolipid metabolism signaling pathways (Fig. 3C). To identify potential molecules associated with lipid metabolism disorders in the SVZ region of adult Seipin KO mice, MALDI-TOF MS was performed. It was shown that the contents of DG, PA, PC, PE, and TG increased significantly (Fig. 3D–F). Oil red O staining showed a significant accumulation of LD in SVZ of adult Seipin KO mice (Fig. 3G), but not in juvenile Seipin KO mice (Additional file 1: Fig. S1). Pearson’s correlation analysis demonstrated a significant negative correlation between LD accumulation area and neurogenesis in mice (Fig. 3H). These results indicated abnormal lipid metabolism in the SVZ region of adult Seipin KO mice, which impaired SVZ neurogenesis and short-term olfactory memory functions.

### AT transplantation restored systemic metabolic homeostasis in Seipin KO mice

**Fig. 4:**
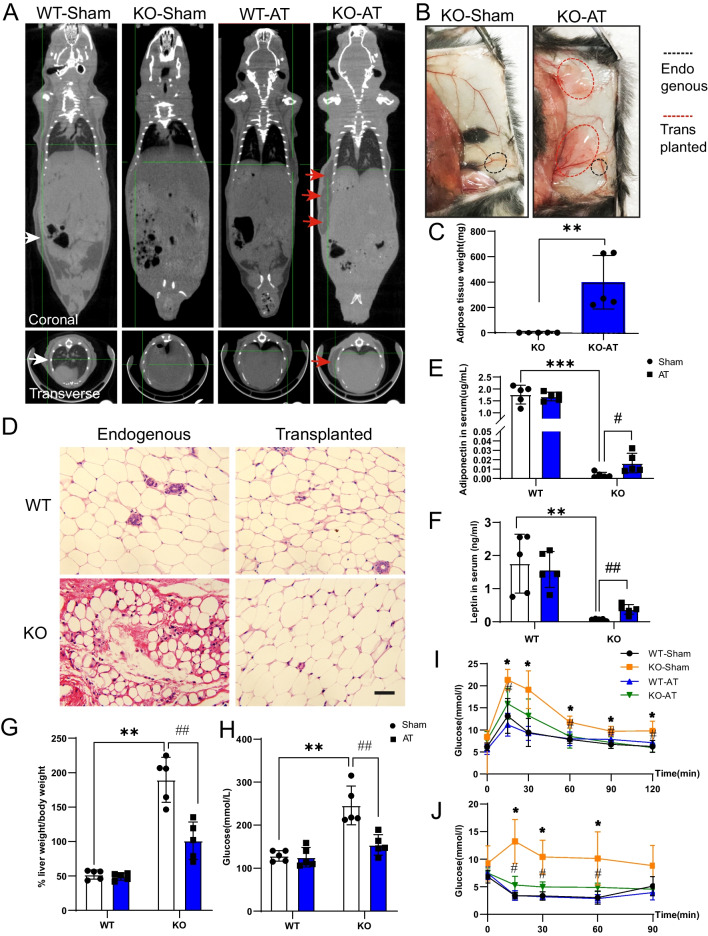
AT transplantation impact on systemic metabolic homeostasis in Seipin KO mice. **A** Representative CT scan images from coronal and transverse views from each group. **B** Representative gross appearance of subcutaneous AT. **C** CT scan showing the weight of surviving AT four months after AT transplantation (n = 5). **p < 0.01 vs. KO-Sham. **D** AT sections stained with HE to show the morphology of transplanted AT. Scale bar = 50 μm. **E** and **F** The serum adiponectin and leptin levels in the Seipin KO mice after AT transplantation (n = 5). **p < 0.01 vs. WT-Sham, ***p < 0.001 vs. WT-Sham, #*p* < 0.05 vs. KO-Sham, ##*p* < 0.01 vs. KO-Sham. **G** and **H** The ratio of liver weight to body weight and fasting blood glucose levels in the Seipin KO mice after AT transplantation (n = 5). **p < 0.01 vs. WT-Sham, ##*p* < 0.01 vs. KO-Sham. **I** and **J** Blood glucose levels after AT transplantation in Seipin KO mice with glucose load (I) and insulin injection (J). n = 5, *p < 0.05 vs. WT-Sham, #*p* < 0.05 vs. KO-Sham. Data were shown as means ± SD

It was reported that AT transplantation could restore the metabolic deficiency of obesity [18]. To check if AT transplantation is feasible on Seipin KO mice, AT was implanted under the dorsal skin of juvenile WT (WT-AT) mice and Seipin KO (KO-AT) mice, respectively, with sham-operation groups (WT-Sham, KO-Sham) as control. Four months later, whole-body CT scans were performed on four groups of adult mice. Coronal scan results showed severe loss of AT and fatty liver in the whole body of KO-Sham mice. In contrast, the dorsal AT distribution of KO-AT mice was comparable with that of WT-Sham mice (Fig. 4A), which was brightly colored, soft and mobile, and significantly vascularized (Fig. 4B). CT scan analysis of adipose volume showed that the surviving fat in KO-AT mice reached 365 mg ± 84 mg after transplantation (Fig. 4C). As shown in Fig. 4D, the morphology of transplanted adipocytes of KO-AT mice was similar to that of WT mice (Fig. 4D). Plasma levels of adiponectin and leptin were significantly increased in KO-AT mice compared with control mice. However, the levels of adiponectin and leptin were decreased in KO mice, indicating that the transplanted AT could perform normal endocrine functions (Fig. 4E and F). The ratio of liver weight to body weight in the KO-AT group was also markedly decreased. (Fig. 4G). Additionally, reduced blood glucose levels, enhanced glucose tolerance, and improved insulin sensitivity were observed in Seipin KO mice after AT transplantation (Fig. 4H–J). These data suggested that AT transplantation could effectively correct systemic metabolic disorders in Seipin KO mice.

### AT transplantation rescued olfactory-related neurobehavior in Seipin KO mice

**Fig. 5:**
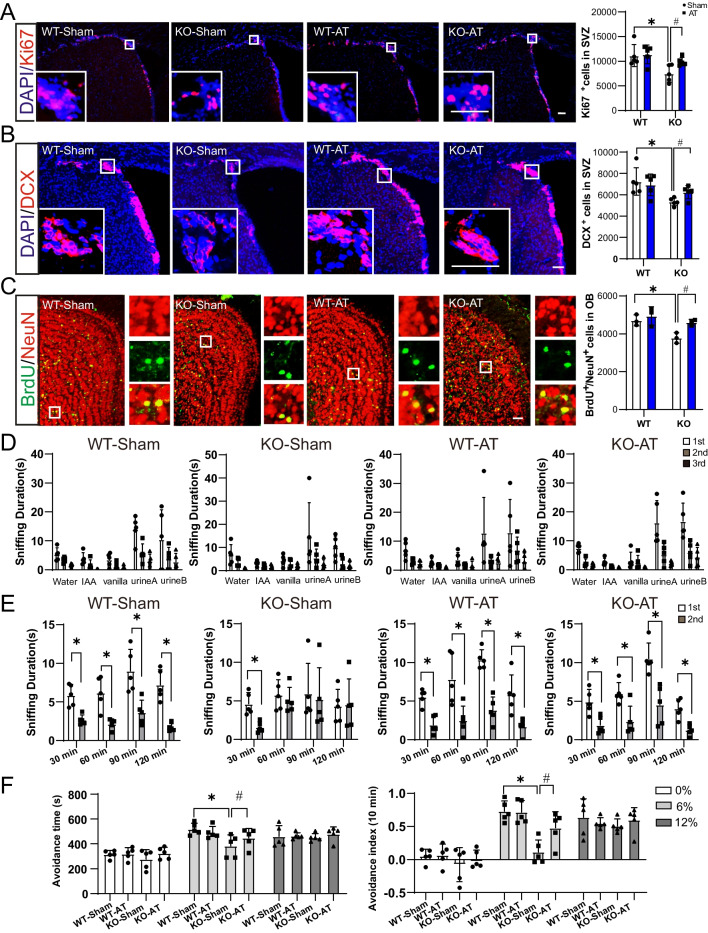
SVZ neurogenesis and neurological function in adult Seipin KO mice after AT transplantation. **A**–**C** Immunofluorescence staining and analysis of Ki67 (A), DCX (B), and BrdU^+^/NeuN^+^ (C) in the SVZ and olfactory bulb granular layer of each group to show neurogenesis of adult Seipin KO mice. n = 5, Scale bar = 50 μm in (A and B); n = 3, Scale bar = 50 μm in (**C**). *p < 0.05 vs. WT-Sham, #*p* < 0.05 vs. KO-Sham. **D** Olfactory habituation/dishabituation assay of Seipin KO and WT mice with or without AT transplantation (n = 5). **E** Short-term olfactory memory test Seipin KO mice before with or without AT transplantation (n = 5). *p < 0.05 vs.1st. **F** Odor avoidance tests in the presence of different doses of nTMT in Seipin KO mice before with or without AT transplantation (n = 5). *p < 0.05 vs. WT-Sham, #*p* < 0.05vs. KO-Sham. Data were shown as means ± SD

After confirming the rescue effect of AT transplantation on systemic metabolism in Seipin KO mice, we further investigate whether AT transplantation improves its neurological deficits. Immunofluorescence staining and behavioral assay were performed in the Seipin KO mice. The number of Ki67^+^ and DCX^+^ in SVZ of KO-Sham group was significantly reduced compared with that of WT-Sham group. AT transplantation restored Ki67^+^ proliferating cells and DCX^+^ neurons in KO-AT mice (Fig. 5A and B). Further, BrdU^+^/NeuN^+^ double staining was applied to determine the number of neonatal neurons in the olfactory bulb granular layer. As shown in Fig. 5C, the number of BrdU^+^/NeuN^+^ neurons in the KO-AT mice was also significantly increased after AT transplantation (Fig. 5C). Olfactory short-term memory test showed that the sniffing duration was significantly reduced in the KO-AT group compared to the KO-Sham group, indicating that AT transplantation could partially restore olfactory-related memory (Fig. 5E), though behavioral tests did not demonstrate a significant difference in olfactory discrimination ability among all groups (Fig. 5D). Additionally, an odor avoidance test was adopted to test the olfactory sensitivity. It showed that when mice were given a solvent (0% nTMT), there was no significant difference of the residence time in the avoidance area. When exposed to 6% nTMT, KO-AT mice demonstrated excellent odor avoidance compared to KO-Sham control. There was no difference between the two groups when nTMT concentrations reached 12%, which might be too strong to dull the sense of smell (Fig. 5F). These results suggested that AT transplantation is beneficial to restore olfactory neurogenesis and olfactory memory of Seipin KO mice.

### AT transplantation reduced the accumulation of DG to alleviate the over-activation of PKCα

**Fig. 6:**
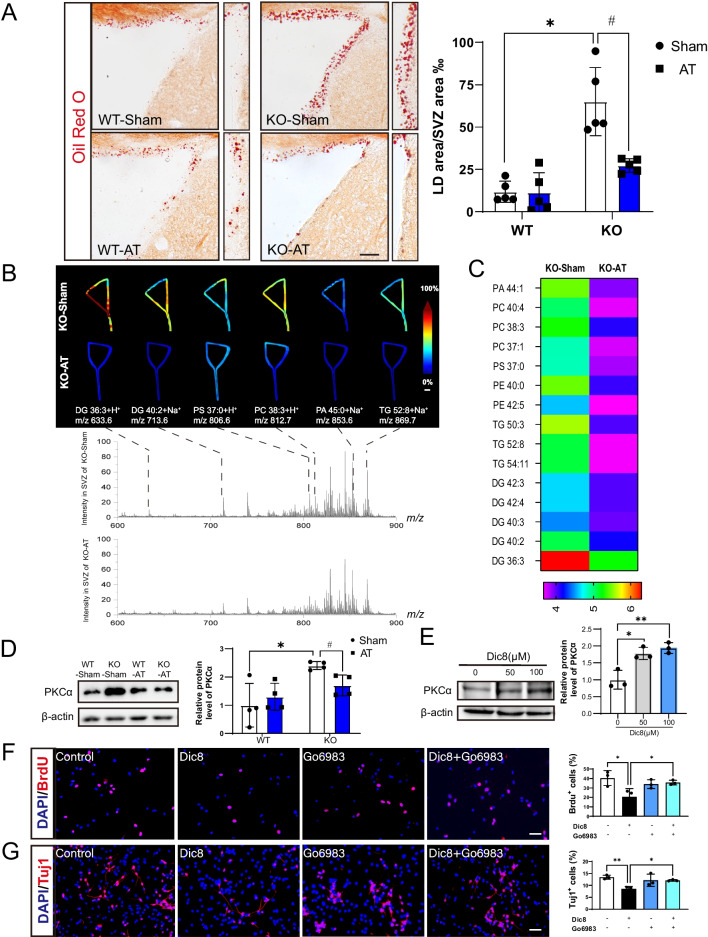
AT transplantation impacting the lipid metabolism and activity of NSCs via the DG-PKCα pathway. **A** Oil Red O staining shows LD in SVZ of Seipin KO and WT mice with or without AT transplantation (n = 5). *p < 0.05 vs. WT-Sham, #*p* < 0.05 vs. KO-Sham. Scale bar = 50 μm. **B** and **C** Imaging mass spectrometry (B) and heatmap (C) analysis of lipid profiles in SVZ of Seipin KO mice with or without AT transplantation to show significantly reduced lipids (n = 3). Scale bar = 100 μm (B). **D** and **E** Western blotting to show the protein expression of PKCα in SVZ of Seipin KO mice before and after AT transplantation (D) and in NSCs derived from SVZ treated with 0, 50, 100 μM DiC8 (E). *p < 0.05 vs. WT-Sham, #*p* < 0.05 vs. KO-Sham, full-length blots are presented in Additional file 3: Fig. S3. **F** and **G** Immunofluorescence analysis for BrdU (F) and Tuj1 (G) to show the impact of DiC8 (100 μM) and PKCα inhibitor on proliferation and differentiation of NSCs derived from SVZ. Scale bar = 50 μm. *p < 0.05. Data were shown as means ± SD

To elucidate the possible mechanism underlying neurobehavioral restoration of AT transplantation in Seipin KO mice, we checked lipid metabolites in the SVZ. Oil red O staining revealed that AT transplantation reduced abnormal lipid accumulation in SVZ of adult Seipin KO mice (Fig. 6A). MALDI-TOF MS results showed that AT transplantation decreased glycerophospholipid, TG and DG content in the SVZ of Seipin KO mice (Fig. 6B and C), indicating that AT transplantation is beneficial to the recovery of lipid homeostasis in SVZ region. PKCα, PKC family member, was reported to be key mediator of lipid metabolism [19]. Hence, we checked the level of PKCα in the SVZ of Seipin KO mice. Gene differential analysis showed that PKCα was up-regulated in SVZ tissues of adult Seipin KO mice (Additional file 2: Fig. S2). Western blotting results confirmed the up-regulation of PKCα in SVZ of adult Seipin KO mice, which was recovered by AT transplantation (Fig. 6D and Additional file 3: Fig. S3). Those results implied that PKCα was involved in the Seipin KO-induced SVZ neurogenesis deficit.

Of the lipid metabolites, DG was one of the most prominently altered. DG was not only the critical intermediate of phospholipids but also served as the second messenger to activate phospholipids and calcium-dependent enzyme PKC and regulate various biological procedures [19, 20]. To check if DG plays crucial role in the activation of PKCα, we applied DG analogue, Dic8, on cultured NSCs. It showed that 12 h of Dic8 treatment significantly increased PKCα level in NSCs (Fig. 6E). BrdU and Tuj1 staining showed that the proliferation and differentiation ability of NSCs were significantly decreased after Dic8 treatment. While PKCα inhibitor, Go6983 reversed phenotypes (Fig. 6F and G). These results suggested that the excessive lipid accumulation, especial DG, might activate PKCα and lead to deleterious effects, which could be ameliorated by PKCα inhibitor.

## Discussion

**Fig. 7:**
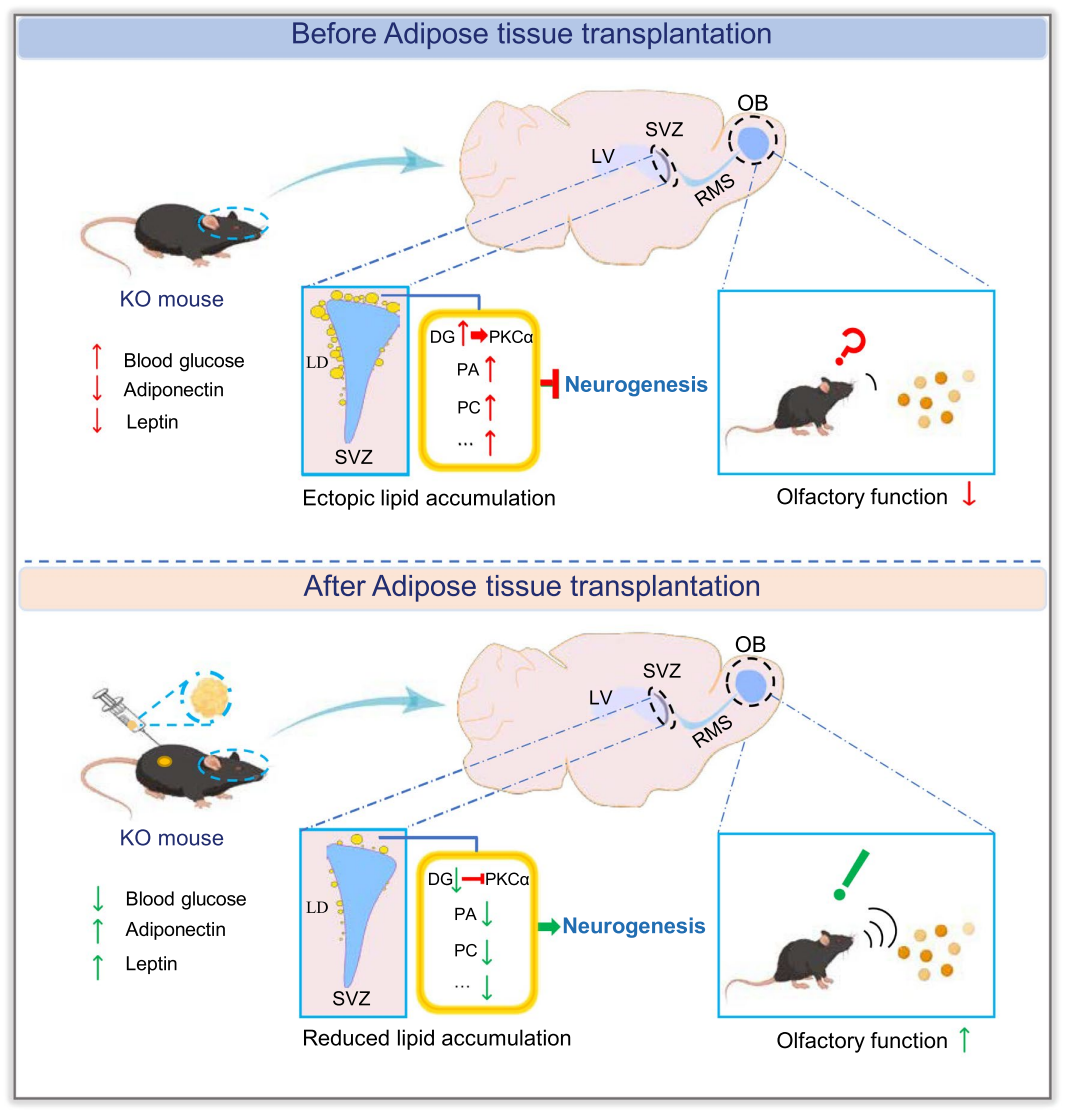
Schematic figure to show the phenotypes of Seipin deficiency on neurogenesis and olfactory function and the rescue effect of adipose transplantation. Seipin deficiency led to lipid metabolites accumulation in SVZ, which compromised neurogenesis and olfactory function. AT transplantation rescued the above phenotypes. This figure was partly generated using Figdraw. OB: olfactory bulb; RMS: rostral migratory stream

Lipid metabolism is a crucial biological process. Aberrant lipid metabolism is implicated in the pathogenesis of various diseases such as metabolic syndrome, neurodegenerative diseases, cancer, and infectious diseases [1, 2]. Seipin was known as a key regulator of lipid homeostasis, and loss of function mutation of Seipin led to not only systemic lipid dysregulation but also neurological deficits [3, 6, 21, 22]. How deficiency of Seipin impairs neuronal development and learning and memory has yet to be fully understood. In the present study, we found that Seipin KO mice displayed abnormal SVZ neurogenesis and olfactory memory dysfunction. MALDI-TOF MS results showed that lipid metabolites such as DG and PA abnormally aggregated in the SVZ region of adult Seipin KO mice. Mechanistically, accumulated DG might further activate PKCα, a lipid metabolism mediator, since in vitro administration of Dic8, an analogue of DG, up-regulated PKCα and inhibited the proliferation and differentiation of NSCs, while PKCα inhibitor could reverse these effects. Furthermore, AT transplantation effectively ameliorated metabolism disorders, attenuated lipid accumulation in the SVZ region, and restored SVZ neurogenesis and olfactory memory in Seipin KO mice. Therefore, modulating the PKCα signaling pathway or AT transplantation may serve as one strategy for the treatment of neurological diseases caused by Seipin deficiency (Fig. 7).

Seipin was abundantly expressed in the central nervous system, and mice with Seipin deficiency exhibited impaired spatial memory and an anxious-depression-like phenotype [5, 6]. It remains controversial about how Seipin deficiency impacts neurobehavior phenotypes. Li et al. reported that Seipin KO suppressed the proliferation and differentiation of hippocampal NSCs, which was responsible for depression-like behavior [6]. Nevertheless, literature reported that Seipin deficiency did not alter the number of hippocampal neuronal cells [23]. In the present study, we found that Seipin deficiency significantly inhibited the proliferation and differentiation of SVZ NSCs in vivo though it was not recapitulated in cultured SVZ NSCs. We speculate that the effects of Seipin deficiency are systemic rather than intrinsic to NSCs. The endocrine environment is apt to influence the neurogenesis of NSCs in SVZ since they efficiently receive peripheral metabolic signals. Other researchers also reported that the proliferation and differentiation of NSCs in the SVZ region of diabetic mice were inhibited [24]. Another study further demonstrated that bilateral adrenalectomy enhanced the proliferation of SVZ NSCs in diabetic mice by reducing corticosterone levels and elevating the expression of brain-derived neurotrophic factors [25]. These observations highlight that metabolic abnormality could directly affect neurogenesis, and maintaining metabolic homeostasis might be an alternative strategy for treating metabolism-related neurological diseases.

Levels and distributions of lipid metabolites are indicators of lipid metabolism [1, 2, 26, 27]. MALDI-TOF MS results demonstrated that glycerophospholipids such as DG, PA, PE, and PC were significantly increased in Seipin KO mice. Transcriptome analysis results also indicated that genes involved in the glycerophospholipid signaling pathway altered substantially in Seipin KO mice. Obesity impaired SVZ adult neurogenesis by promoting the release of inflammatory cytokines and causing the accumulation of LDs in SVZ [12]. Similarly, lipid accumulation was found in the hypothalamus of obese mice [28, 29]. It indicated that lipid metabolites directly contributed to the pathogenesis of neurological diseases [30, 31].

DG was an intermediate molecule of glycerophospholipid metabolism and participated in various biological processes [32, 33]. In AD and Sjogren–Larsson syndrome patients, elevated DG was reported to be associated with cognitive or intellectual impairment [30, 31, 34]. In the current study, we found a significant elevation of DG in the SVZ of Seipin KO mice. Moreover, treatment with Dic8, a DG analogue, effectively suppressed the proliferation of NSCs in vitro, indicating that the excessive accumulation of DG might account for the impaired activity of NSCs. One study reported that abnormal fatty acid metabolism happened in the SVZ region of an AD mouse brain, leading to impaired proliferation of NSCs, and inhibition of oleic acid synthesis effectively rescued the defects [13]. The presence of aberrant lipid metabolites may be correlated with the occurrence of diseases, and detecting them would benefit the diagnosis of diseases. To be noted, abnormality of different lipid metabolites might render distinct disease, and precise analysis of it is necessary.

In this study, we also attempted to restore systemic lipid metabolic homeostasis and investigated its rescue effect on neurogenesis [18, 35]. As well known, AT not only served as an organ for energy storage but also as the endocrine organ to regulate metabolic homeostasis [36, 37]. AT transplantation has been widely implemented in plastic surgery and burn treatment due to its reproducibility, ease of obtaining in large quantities and less damage to the donor [37–39]. Previous research showed that subcutaneous AT transplantation restored peripheral metabolic balance and improved hepatic steatosis and kidney function of Seipin KO mice [22, 40]. Our study indicated AT transplantation restored SVZ neurogenesis and olfactory function of Seipin KO mice accompanied by restoration of systemic metabolic balance. Mechanistically, previous studies had shown that the adiponectin and leptin derived from AT could exert neuroprotective effects by promoting adult neurogenesis [41, 42]. Interestingly, adipose tissue-derived extracellular vesicles (EVs), instead of AT, were reported to stimulate neurogenesis of AD mice and rescue memory defects [43]. All these results suggested that AT transplantation represented a potential therapeutic strategy for lipodystrophic disorders and their associated neurological deficits.

Lipid metabolism is precisely regulated to maintain homeostasis in the body. PKCα is one key mediator of lipid metabolism, and alteration of it is implicated in neurological diseases. PKCα possessed one C1 domain with a higher affinity for DG, a metabolite of lipid metabolism [19]. It was proved that activation of PKCα by DG analogue induced the apoptosis of cerebellar granular cells and inhibited the differentiation of neural precursor cells [44, 45]. Moreover, PKCα was up-regulated in AD brain and PKCα inhibitors can block the endocytosis and synaptic inhibition of glutamate receptors produced by Aβ [46, 47]. Here, we found that the expression of PKCα was increased in the SVZ of Seipin KO mice. In vitro, the addition of DG analogue, Dic8, suppressed the proliferation and differentiation of NSCs, whereas Go6983, an inhibitor of PKCα, reversed the inhibitory effect of Dic8 on NSCs. Our study and others suggest that PKCα plays a crucial role in mediating diseases caused by lipid metabolism disorders. PKCα may be one of the potential targets for the intervention of neurological disorders related to abnormal lipid metabolism.

However, there are still some limitations in the present study. For example, the in vivo effect of PKCα inhibitor in Seipin KO mice remains to be explored. Whether other cell types in the brain, such as microglia and astrocytes, are involved in the defective neurogenesis phenotype induced by lipid disorders caused by Seipin depletion remains worthy of further investigation.

## Conclusion

Overall, our study demonstrates for the first time that suppressed SVZ neurogenesis and impaired olfactory function were found in Seipin KO mice, which is closely related to systemic lipid metabolism and ectopic lipid accumulation in SVZ region disorders. AT transplantation could effectively relieve metabolic disorders of Seipin KO mice and restore the SVZ neurogenesis and olfactory memory. Mechanistically, PKCα, a lipid metabolism mediator, was up-regulated in the SVZ region of Seipin KO mice, which may be activated by DG. Furthermore, in vitro NSCs culture experiment indicates that DG analogue treatment can mimic this process. Overall, our work demonstrates that AT transplantation and PKCα inhibition may represent two effective intervention ways for lipid dysregulation-involved neurological deficits.

### Supplementary Information

**Additional file. 1: Fig. S1**. LD accumulation was not significant in SVZ of juvenile KO mice. Oil Red O detection of LD in the SVZ of the juvenile brain (n = 5), Scale bar = 50 μm. Data were shown as means ± SD. ns, not significant vs. juvenile WT mice.

**Additional file 2: Fig. S2**. PKCα is associated with lipid metabolism pathways. Chordal diagram showed the relationship between KEGG enrichment pathways and genes, and were shown in different colors.

**Additional file 3: Fig. S3**. The full length of original blots. The blots shown in Figure 6D and E were highlighted with red boxes.

## Data Availability

The datasets generated and analyzed during the current study are available from the corresponding author on reasonable request. The raw sequencing data presented in this paper have been deposited at Sequence Read Archive (SRA) under accession number PRJNA974405.

## Abbreviations

AD: Alzheimer’s disease
AT: Adipose tissue
BBB: Blood–brain barrier
bFGF: Basic fibroblast growth factor
CGL2: Type 2 congenital generalized lipodystrophy
CT: Computed tomography
DEGs: Differentially expressed genes
DG: Diacylglycerol
EGF: Epidermal growth factor
GO: Gene Ontology
HE: Hematoxylin and eosin
IAA: Isoamyl acetate
IR: Insulin resistance
ITO: Indium tin oxide
KO: Knockout
KEGG: Kyoto Encyclopedia of Genes and Genomes
LD: Lipid droplets
LV: Lateral ventricle
MALDI-TOF MS: Matrix-assisted laser desorption ionization time-of-flight mass spectrometry
NSCs: Neural stem cells
nTMT: Non-dehydrogenated 2, 4, 5-trimethylthiazole
OB: Olfactory bulb
PA: Phosphatidic acid
PC: Phosphatidylcholine
PD: Parkinson’s disease
PE: Phosphatidylethanolamine
PFA: Paraformaldehyde
PKC: Protein kinase C
PET: Positron emission tomography
PS: Phosphatidylserine
RMS: Rostral migratory stream
RT: Room temperature
SD: Standard deviations
SVZ: Subventricular zone
TG: Triglyceride
WT: Wild type
