# Supplementary figures and images for: Adipose transplantation improves olfactory function and neurogenesis via PKCα-involved lipid metabolism in Seipin Knockout mice
Source: Stem Cell Res Ther. 2023 Sep 7;14:239. doi: 10.1186/s13287-023-03463-9 (PMC10483743; doi:10.1186/s13287-023-03463-9)

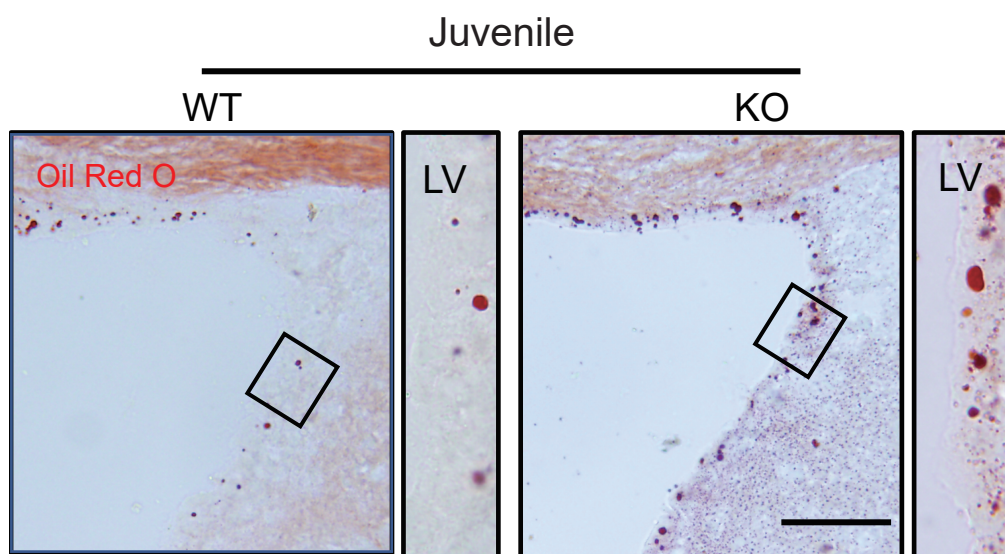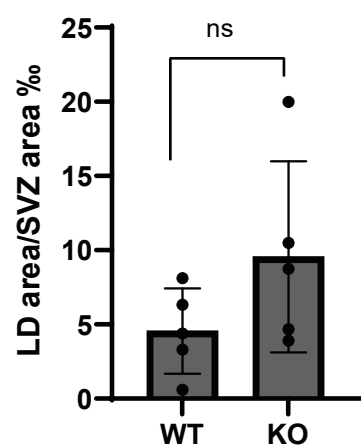

Supplement: Supplementary file 1 — Additional file. 1: Fig. S1. LD accumulation was not significant in SVZ of juvenile KO mice. Oil Red O detection of LD in the SVZ of the juvenile brain (n = 5), Scale bar = 50 μm. Data were shown as means ± SD. ns, not significant vs. juvenile WT mice. [file 13287_2023_3463_MOESM1_ESM.pdf]

The Full-Length blots for Figure 6D

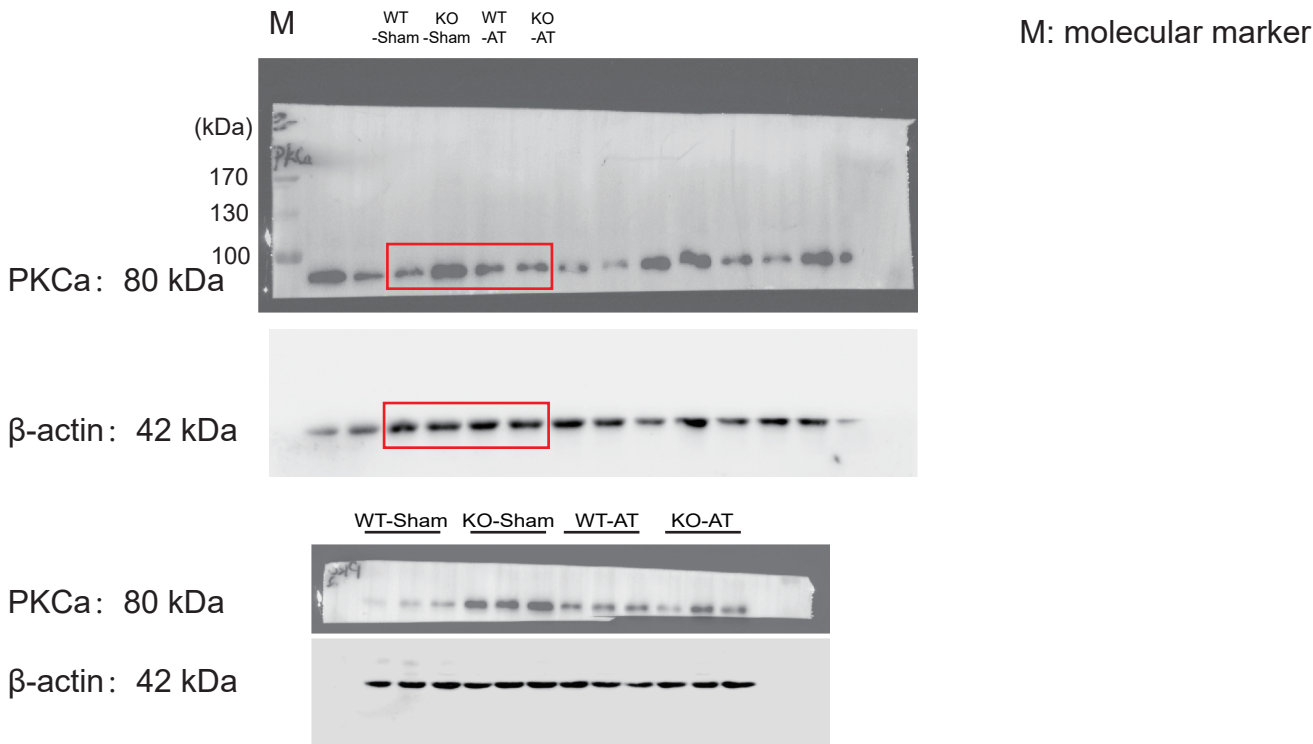

The Full-length blots of Figure 6E

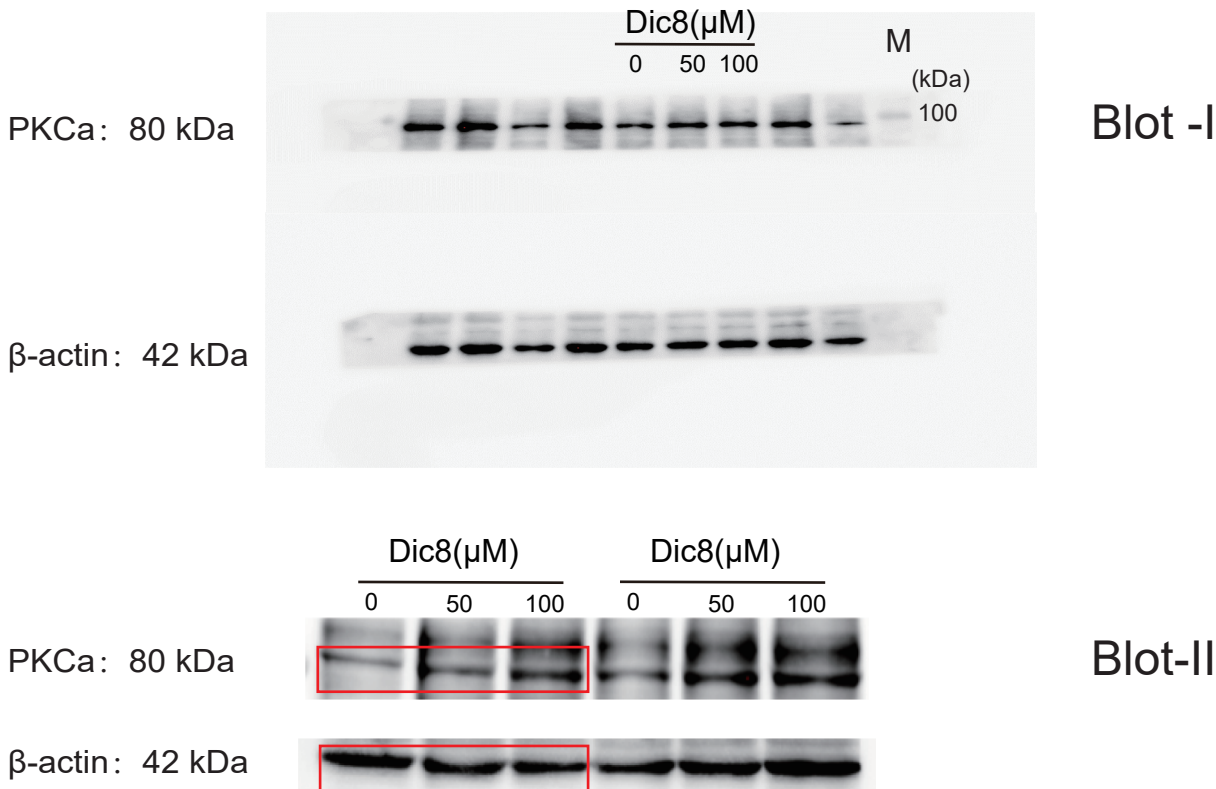

Supplement: Supplementary file 3 — Additional file 3: Fig. S3. The full length of original blots. The blots shown in Figure 6D and E were highlighted with red boxes. [file 13287_2023_3463_MOESM3_ESM.pdf]
