# Supplementary material for: Adipose transplantation improves olfactory function and neurogenesis via PKCα-involved lipid metabolism in Seipin Knockout mice
Source: Stem Cell Res Ther. 2023 Sep 7;14:239. doi: 10.1186/s13287-023-03463-9 (PMC10483743; doi:10.1186/s13287-023-03463-9)
Supplement: Supplementary file 2 — Additional file 2: Fig. S2. PKCα is associated with lipid metabolism pathways. Chordal diagram showed the relationship between KEGG enrichment pathways and genes, and were shown in different colors. [file 13287_2023_3463_MOESM2_ESM.pdf]

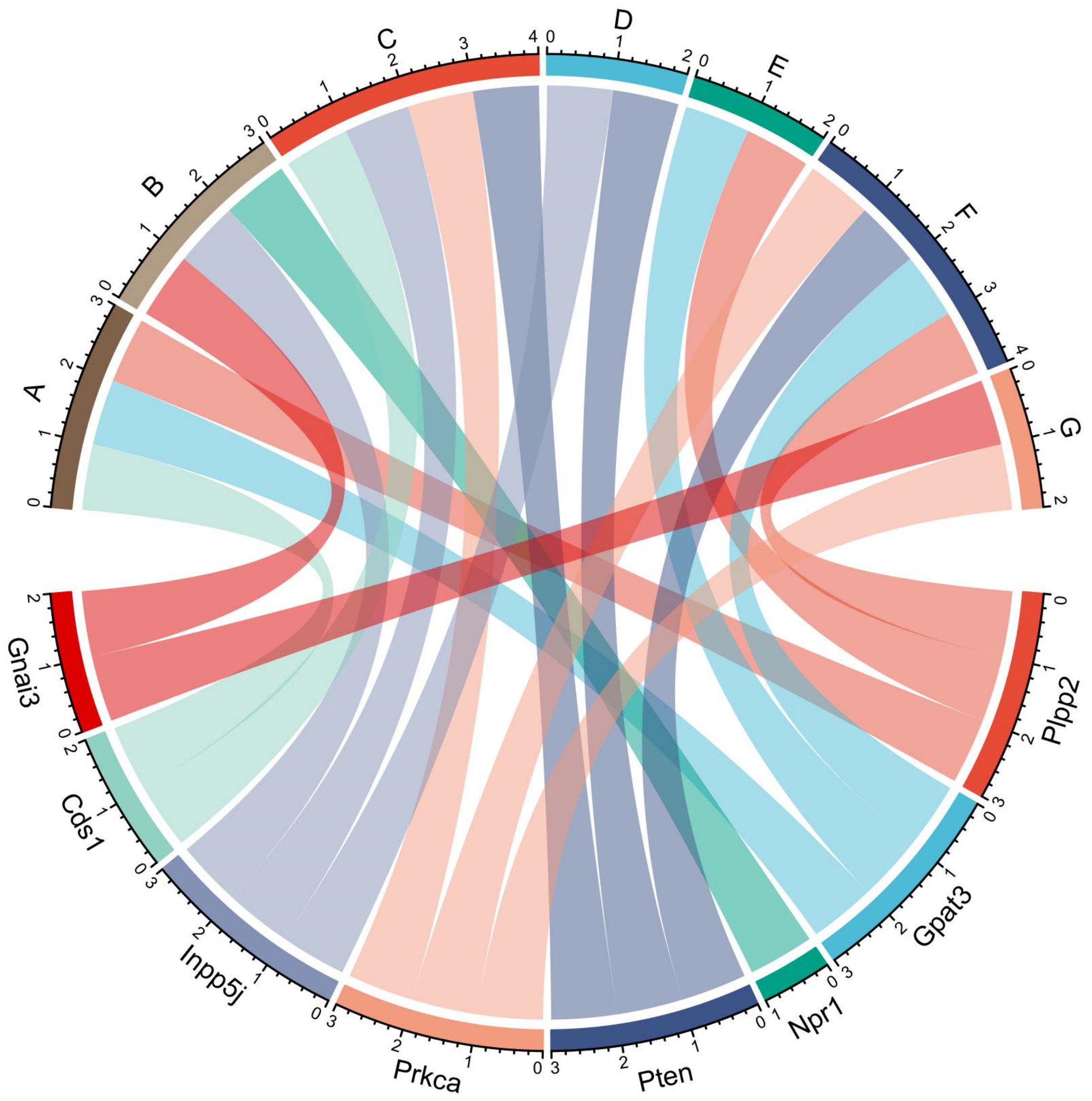

A Glycerophospholipid metabolism

B Regulation of lipolysis in adipocytes

C Phosphatidylinositol signaling system

D Inositol phosphate metabolism

E Glycerolipid metabolism

F Phospholipase D signaling pathway

G Sphingolipid signaling pathway
